# Do Major Roads Reduce Gene Flow in Urban Bird Populations?

**DOI:** 10.1371/journal.pone.0077026

**Published:** 2013-10-18

**Authors:** Shuping Zhang, Mingli Suo, Shenglin Liu, Wei Liang

**Affiliations:** 1 College of Life and Environment Sciences, Minzu University of China, Beijing, China; 2 Ministry of Education Key Laboratory for Tropical Animal and Plant Ecology, College of Life Sciences, Hainan Normal University, Haikou, China; CNRS, Université de Bourgogne, France

## Abstract

**Background:**

Although the negative effects of roads on the genetics of animal populations have been extensively reported, the question of whether roads reduce gene flow in volant, urban bird populations has so far not been addressed. In this study, we assess whether highways decreased gene flow and genetic variation in a small passerine bird, the tree sparrow (*Passer montanus*).

**Methodology:**

We assessed genetic differences among tree sparrows (*Passer montanus*) sampled at 19 sites within Beijing Municipality, China, using 7 DNA microsatellites as genetic markers.

**Results:**

AMOVA showed that genetic variation between sites, between urban and rural populations, and between opposite sides of the same highway, were very weak. Mantel tests on all samples, and on urban samples only, indicated that the age and number of highways, and the number of ordinary roads, were uncorrelated with genetic differences (*F*_ST_) among tree sparrows from different urban sites. Birds sampled at urban sites had similar levels of genetic diversity to those at rural sites. There was, however, evidence of some weak genetic structure between urban sites. Firstly, there were significant genetic differences (*F*_ST_) between birds from opposite sides of the same highway, but no significant *F*_ST_ values between those from sites that were not separated by highways. Secondly, birds from eleven urban sites had loci that significantly deviated from the Hardy–Weinberg equilibrium but no such deviation was found in birds from rural sites.

**Conclusion:**

We cannot, therefore, conclusively reject the hypothesis that highways have no effect on the gene flow of tree sparrow populations. Furthermore, since the significance of these results may increase with time, we suggested that research on the influence of highways on gene flow in urban bird populations needs to be conducted over several decades.

## Introduction

The ecological effects of roads have long been identified [1]. The most commonly reported impacts of roads on animal populations include habitat loss, edge effects, genetic isolation, road mortality and increased human access [1]–[5]. In recent decades, there has been growing interest in how roads act to reduce gene flow between animal populations [6]–[8]. Reduced dispersal can lead to the loss of genetic diversity through genetic drift [9], which in turn is likely to increase population extinction rates through inbreeding [10], [11]. Negative genetic effects of roads on various species, ranging from crickets and ground beetles to amphibians and mammals, have been reported [12]–[15].

Previous research on the influence of roads on gene flow in animal populations has focused on flightless species [8], to date, no study has addressed the question of whether roads also affect gene flow in volant taxa such as passerine birds. Generally, wider roads with greater volumes of high-speed traffic have a greater effect on animal populations than smaller, less travelled roads [16], [17]. Several studies have confirmed that highways play a role in the decline of bird populations in species such as sparrows and blackbirds [18]–[22]. There is also evidence that proximity to highways decreases the probability of occurrence of both forest and urban birds [23], [24]. In light of these facts, there is growing concern that highways could reduce dispersal and gene flow in urban bird populations. In this study, we assess whether highways decreased gene flow and genetic variation in a small passerine bird, the tree sparrow (*Passer montanus*).

More specifically, we test the following predictions:

1. Genetic differences between tree sparrows at different sites are positively correlated with the number, or age, of multilane highways between sampling sites.
2. Genetic differences between samples from opposite sides of the same highway are greater than those between samples from the same side of a highway.
3. Urban tree sparrows display greater inter-site genetic differentiation and lower genetic diversity than rural tree sparrows.

## Methods

### Study species

The tree sparrow is a relatively sedentary species that occurs in a variety of habitats throughout China [25], [26]. Tree sparrows have relatively small home ranges with an estimated radius of 100 to 300 m [26], [27]. In a previous study, we found that virtually no tree sparrows live alongside major roads in urban Beijing [24]. For these reasons, we chose tree sparrows to examine the effect of urban highways on the population genetics of a volant, but relatively sedentary, bird species.

### Sample collection

**Figure 1:**
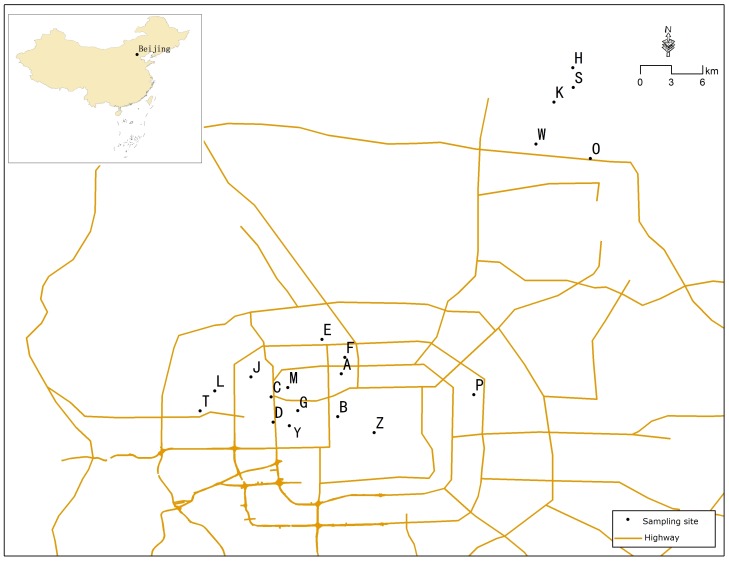
Map showing sites at which blood samples were collected from tree sparrows in Beijing and outlying rural sites, China.

We collected blood samples from tree sparrows at 14 sites in urban Beijing which has a dense road network with six concentric ring highways and ten intercity highways (Figure 1). We also collected samples from five rural sites outside Beijing. All samples were collected during May 2010; at this time of year juveniles have not yet fledged which reduced the possibility of catching close relatives. At least 20 adult sparrows were captured in mist-nets at each sample site. Birds were removed from mist-nets and processed as quickly as possible to minimize stress; all captured tree sparrows were released alive after blood sampling. A small blood sample (50–100 µl) was collected from each bird immediately after its capture by puncturing a brachial wing vein with a disinfected 23 G needle and collecting the blood that exuded from the puncture site into heparized microcapillary tubes [28]. The skin around the puncture site was disinfected with medical alcohol before and after puncturing. Pressure was applied to the puncture site for 1 min with an alcohol-soaked cotton wool swab to staunch bleeding. Blood samples were transported to the lab under refrigeration and then stored at −20°C until used in analyses.

All samples were collected with the permission of the Beijing Forestry Bureau. Experimental procedures conformed to the relevant Chinese laws and had the approval of Hainan Normal University's Animal Research Ethics Committee (see also [29]). In addition, all procedures followed standard protocols, such as the ARRIVE guidelines for reporting animal research [30].

### Molecular Analysis

DNA samples were extracted from blood samples following the procedure described by Griffith et al. [31]. Seven unlinked and highly polymorphic microsatellite loci were used in genetic analysis (Table 1). For each sample, PCR was carried out in a PTC-200 thermal cycler using the following protocol: an initial hot start for 2 min at 94°C, followed by 35 cycles of 20 s at 94°C, 30 s at the annealing temp (Table 1), and 60 s at 72°C. The fragment sizes were analyzed using an ABI310 gene sequencer. The PCR products were mixed with size standard (500 bp ROX-labelled fragments) and deionized formamide at the ratio of 1∶12∶0.5 and run on the sequencer. Microsatellite allele sizes were scored using genescan software after calibration with the internal size standard.

**Table 1:** Microsatellite DNA loci used in the present study.

Locus	Reference	Expected PCR product length (bp)	PCR annealing temperature (°C)
HrU5-A	Primmer et al. 1995	141–180	50°C
Pdoμ3	Griffith et al., 1999	109–153	54°C
Pdoμ5	Griffith et al., 1999	210–268	65°C
Pdo10	Griffith et al., 2007	113–147	60°C
Ase18	Richardson et al. 2000	185–249	54°C
WBSW11	McRae & Amos 1999	177–283	55°C
Fhu2	Primmer et al. 1996b	128–160	58°C

### Data Analysis

#### Population differentiation

Pairwise multilocus *F*_ST_ values were used to estimate genetic differentiation between populations. *F*_ST_ values were calculated in *Arlequin* 3.0. Three hierarchical analyses of molecular variance (AMOVA; [32]) using different grouping methods were conducted in *Arlequin* 3.0. Method A treated all the samples obtained from each site as a single group, Method B treated samples collected on the same side of the same highway as a group and Method C divided all samples into urban and rural groups. Three hierarchical levels were analyzed for all three Methods:1)variance among groups, 2) variance among individuals within group, 3)variance within individuals. The significances of all F statistics values were tested via 16000 permutations.

### Mantel test

The correlation between *F*_ST_, geographical distance, number of highways, highway age and number of roads between sample sites were tested using a Mantel test carried out in Fstat. Because urban highways have much more traffic than those in rural areas, highways would be expected to be a greater barrier to gene flow in urban than rural tree sparrow habitat. To determine if this was the case we performed two Mantel tests: one on all samples, and a second on urban samples only. By means of a permutation procedure, the significance of the partial regressions between the pairwise *F*_ST_ values and the following four matrices were tested: geographical distance as measured on a map, number of highways, total age of highways in years and number of roads.

### Genetic diversity within populations

The observed and expected heterozygosities were estimated for each locus from each sample site. Significant departures from the Hardy–Weinberg equilibrium were detected applying tests which were carried out with 1000000 steps in the Markov chain and 5000 dememorization steps [33]. A sequential Bonferroni correction for multiple tests [34] was applied to the data from each sample site. The linkage disequilibria between all pairs of loci were tested with a Likelihood-ratio test [35]. All the above statistical analyses were conducted in Arlequin 3.0. Allelic richness was calculated using HP-rare [36] which uses rarefaction to correct for sampling error.

## Results

### Genetic diversity

All seven microsatellite loci were polymorphic in all samples, with total allelic richness ranging between 4.972 and 5.586 (Table 2). Total observed heterozygosity varied from 0.778 to 0.849, and the total expected heterozygosity varied from 0.835 to 0.877 (Table 2). There was no significant difference in genetic diversity between urban and rural tree sparrows (Mann-Whitney U test, A: *P* = 0.459, Ho: *P* = 0.622, He: *P* = 0.559). However, eleven of the 14 urban sites had heterozygosity values significantly different from those expected under Hardy–Weinberg equilibrium (*P*<0.05) (Table 2). No such departure from the Hardy–Weinberg equilibrium was found in samples from the rural sites (Table 2).

**Table 2:** Genetic variability at seven microsatellite loci in tree sparrows from 19 different sites in urban and rural Beijing, China.

		locus name, repeat motif							
		HrU5-A	Pdoμ3	Fhu2	Pdo10	Ase18	Pdoμ5	WBSW11	All
urban sites									
A	A	4.561	5.201	3.478	5.261	6.699	6.317	6.011	5.361
n = 28	H_O_	0.714	0.786	0.607	0.893	0.929	0.857	0.836	0.803
	H_E_	0.809	0.869	0.701	0.860	0.948	0.929	0.905	0.860
B	A	4.904	4.392	3.548	4.409	7.045	6.223	6.263	5.255
n = 28	H_O_	0.733	0.733	0.867	0.600	1.000	0.933	0.700*	0.795
	H_E_	0.839	0.805	0.690	0.775	0.963	0.917	0.929	0.845
C	A	4.906	4.739	3.424	4.495	6.870	5.853	6.339	5.232
n = 30	H_O_	0.903	0.902	0.767	0.601	0.933	0.905	0.907	0.845
	H_E_	0.844	0.825	0.708	0.785	0.955	0.904	0.926	0.849
D	A	5.083	4.889	3.513	4.480	6.727	6.176	6.710	5.368
n = 24	H_O_	0.708	0.875	0.708	0.625	0.958	0.917	0.833	0.803
	H_E_	0.843	0.853	0.711	0.786	0.947	0.920	0.948	0.858
E	A	5.013	4.988	3.457	4.615	6.536	6.623	6.549	5.397
n = 26	H_O_	0.885	0.846	0.846	0.731	0.923	0.769*	0.877	0.839
	H_E_	0.841	0.859	0.698	0.785	0.935	0.945	0.937	0.850
F	A	5.017	4.869	3.767	4.977	6.725	6.447	6.536	5.477
n = 29	H_O_	0.655*	0.897	0.655	0.724	0.964	0.966	0.759*	0.802
	H_E_	0.851	0.839	0.709	0.843	0.950	0.936	0.932	0.866
G	A	4.903	4.859	3.581	3.576	5.862	6.164	5.859	4.972
n = 25	H_O_	0.867	0.800	0.733	0.600	0.933	0.933	0.767	0.804
	H_E_	0.837	0.841	0.747	0.715	0.899	0.917	0.890	0.835
J	A	5.090	5.089	3.712	4.578	6.692	6.120	6.278	5.366
n = 37	H_O_	0.892	0.730	0.622	0.676	0.919	0.838	0.814	0.784
	H_E_	0.855	0.863	0.732	0.801	0.946	0.918	0.923	0.861
L	A	4.982	4.416	3.513	5.267	6.443	6.086	6.015	5.246
n = 29	H_O_	0.724	0.793	0.621	0.862	0.857	0.929	0.790	0.796
	H_E_	0.845	0.793	0.730	0.858	0.936	0.913	0.884	0.851
M	A	4.911	4.937	3.539	5.362	6.569	5.949	6.875	5.449
n = 39	H_O_	0.795	0.897	0.769	0.897	0.923	0.872	0.790*	0.849
	H_E_	0.841	0.848	0.737	0.867	0.941	0.900	0.956	0.866
P	A	4.724	5.069	3.394	4.659	6.685	5.915	5.827	5.182
n = 28	H_O_	0.857	0.571*	0.821	0.786	0.857	0.821	0.736	0.778
	H_E_	0.832	0.849	0.714	0.823	0.946	0.904	0.897	0.852
T	A	4.813	5.165	3.921	5.422	6.602	6.601	5.543	5.438
n = 26	H_O_	0.692*	0.846	0.808	0.885	0.923	0.885	0.692*	0.818
	H_E_	0.829	0.864	0.768	0.878	0.941	0.942	0.864	0.869
Y	A	4.121	4.750	4.362	5.150	6.781	6.447	6.582	5.456
n = 35	H_O_	0.800	1.000	0.733	0.600*	0.867	0.933	0.867	0.828
	H_E_	0.761	0.816	0.800	0.857	0.952	0.938	0.943	0.866
Z	A	4.721	4.970	3.598	4.839	6.569	6.522	6.390	5.373
n = 33	H_O_	0.758	0.758	0.606	0.697*	0.879	0.939	0.818	0.779
	H_E_	0.825	0.848	0.724	0.826	0.942	0.938	0.930	0.861
Rural sites									
H	A	5.105	5.179	3.627	5.341	6.848	6.245	6.755	5.586
n = 35	H_O_	0.942	0.809	0.714	0.771	0.885	0.857	0.809	0.826
	H_E_	0.854	0.861	0.738	0.866	0.954	0.925	0.948	0.878
K	A	4.708	5.046	3.586	4.354	6.656	6.416	6.123	5.270
n = 29	H_O_	0.678	0.862	0.758	0.724	0.983	0.896	0.851	0.822
	H_E_	0.794	0.854	0.722	0.767	0.944	0.935	0.904	0.845
S	A	5.366	4.958	3.622	4.336	6.504	6.226	6.495	5.359
n = 54	H_O_	0.870	0.824	0.716	0.701	0.888	0.981	0.832	0.830
	H_E_	0.871	0.851	0.734	0.751	0.937	0.924	0.936	0.857
W	A	5.116	5.082	3.693	4.876	6.607	6.282	6.573	5.461
n = 35	H_O_	0.812	0.765	0.763	0.706	0.835	0.860	0.809	0.792
	H_E_	0.861	0.883	0.798	0.825	0.904	0.942	0.927	0.877
O	A	5.057	5.079	3.549	4.527	6.624	6.216	6.483	5.362
n = 32	H_O_	0.798	0.871	0.758	0.764	0.905	0.853	0.801	0.821
	H_E_	0.894	0.854	0.722	0.758	0.931	0.916	0.904	0.854

* heterozygosity values significantly different from those expected under the Hardy–Weinberg equilibrium (*P*<0.05).

### Population differentiation

In 171 pairwise tests for genetic differentiation between sample sites, 17 (9.9%) were significant at the *P*<0.05 level, 15 (8.7%) were significant at the *P*<0.01 level and 3 (1.8%) were significant at the *P*<0.001 level (Table 3). Therefore, a total of 20% of paired sites had significant genetic differences. All significant genetic differences occurred between birds from sites on opposite sides of the same highway. There were no significant genetic differences between birds from sites with no highway between them. AMOVA revealed that most (99%) of the variance was explained by within-group variation (Table 4).

**Table 3:** Pairwise *F*st estimates (below the diagonal) and *P* values of G-tests of pairwise differentiation between tree sparrows from different sites in Beijing (above the diagonal), China.

	A	B	C	D	E	F	G	J	L	M	P	T	Y	Z	H	K	S	O	W
A	-	n.s.	n.s.	n.s.	**	n.s.	*	n.s.	**	*	n.s.	**	n.s.	n.s.	n.s.	n.s.	**	n.s.	n.s.
B	0.0140	-	n.s.	n.s.	n.s.	n.s.	n.s.	n.s.	n.s.	n.s.	n.s.	n.s.	n.s.	**n.s.**	n.s.	n.s.	*	n.s.	n.s.
C	0.0098	0.0054	-	**n.s.**	*	n.s.	n.s.	**n.s.**	n.s.	n.s.	n.s.	*	n.s.	n.s.	n.s.	n.s.	n.s.	n.s.	n.s.
D	0.0000	0.0091	**0.0030**	-	n.s.	n.s.	n.s.	**n.s.**	*	n.s.	n.s.	*	n.s.	n.s.	n.s.	n.s.	n.s.	n.s.	n.s.
E	0.0212	0.0149	0.0125	0.0102	-	n.s.	**	*	***	***	**	**	n.s.	n.s.	*	**	**	*	*
F	0.0073	0.0058	0.0071	0.0027	0.0075	-	n.s.	n.s.	n.s.	n.s.	n.s.	n.s.	n.s.	n.s.	n.s.	n.s.	*	n.s.	n.s.
G	0.0168	0.0094	0.0089	0.0097	0.0251	0.0060	-	n.s.	n.s.	n.s.	n.s.	n.s.	**n.s.**	n.s.	n.s.	n.s.	n.s.	n.s.	n.s.
J	0.0092	0.0093	**0.0028**	**0.0035**	0.0105	0.0059	0.0119	-	*	n.s.	n.s.	*	n.s.	n.s.	n.s.	n.s.	n.s.	n.s.	n.s.
L	0.0180	0.0064	0.0069	0.0110	0.0209	0.0078	0.0097	0.0100	-	n.s.	n.s.	**n.s.**	n.s.	n.s.	n.s.	**	*	n.s.	*
M	0.0122	0.0058	0.0052	0.0014	0.0159	0.0079	0.0112	0.0068	0.0028	-	n.s.	*	n.s.	n.s.	n.s.	***	**	n.s.	**
P	0.0100	0.0128	0.0008	0.0058	0.0170	0.0099	0.0082	0.0047	0.0075	0.0038	-	n.s.	n.s.	n.s.	n.s.	n.s.	n.s.	n.s.	n.s.
T	0.0185	0.0107	0.0106	0.0111	0.0156	0.0014	0.0090	0.0129	**0.0021**	0.0100	0.0099	-	n.s.	n.s.	n.s.	**	**	n.s.	**
Y	0.0019	0.0074	0.0011	0.0022	0.0135	0.0010	**0.0043**	0.0006	0.0016	0.0006	0.0037	0.0088	-	n.s.	n.s.	n.s.	n.s.	n.s.	n.s.
Z	0.0068	**0.0045**	-0.0009	0.0012	0.0092	0.0029	0.0106	0.0010	0.0043	0.0034	0.0004	0.0048	-0.0029	-	n.s.	n.s.	n.s.	n.s.	n.s.
H	0.0050	0.0058	0.0002	0.0011	0.0122	0.0017	0.0049	0.0013	0.0027	0.0009	0.0176	0.0057	0.0037	0.0007	-	**n.s.**	**n.s.**	**n.s.**	**n.s.**
K	0.0063	0.0142	0.0076	0.0084	0.0180	0.0103	0.0120	0.0161	0.0148	0.0176	0.0028	0.0184	0.0089	0.0088	**0.0073**	**-**	**n.s.**	**n.s.**	**n.s.**
S	0.0135	0.0170	0.0044	0.0015	0.0157	0.0106	0.0117	0.0063	0.0123	0.0100	0.0047	0.0124	0.0076	0.0011	**0.0039**	**0.0088**	-	**n.s.**	**n.s.**
O	0.0042	0.0051	0.0007	0.0009	0.0128	0.0020	0.0043	0.0019	0.0021	0.0011	0.0153	0.0049	0.0007	0.0009	**0.0027**	**0.0056**	**0.0029**	**-**	**n.s.**
W	0.0074	0.0151	0.0072	0.0059	0.0134	0.0112	0.0132	0.0159	0.0131	0.0169	0.0031	0.0179	0.0085	0.0076	**0.0036**	**0.0021**	**0.0032**	**0.0013**	-

* *P*<0.05;

** *P*<0.01;

*** *P*<0.001;

n.s.: no significant difference; The *F*st and significance of paired sites with no highway between them are shown in bold.

**Table 4:** Genetic variance components and hierarchical F statistics for tree sparrows from Beijing.

Grouping method	Source of variation	*d.f.*	Sum of squares	Variance component	Percentage of variation	F statistics	*P*
A	among groups	18	88.642	0.016	0.46%	0.005(*F*_GT_)	0.013
	among individuals within group	583	2339.103	0.591	17.21%	0.173(*F*_IG_)	0.016
	within individuals	602	1703.219	2.829	82.33%	0.177(*F*_IT_)	0.017
B	among groups	1	6.427	0.007	0.15%	0.002(*F*_GT_)	0.031
	among individuals within group	600	2422.217	0.604	17.51%	0.175(*F*_IG_)	0.015
	within individuals	602	1703.219	2.829	82.34%	0.177(*F*_IT_)	0.018
C	among groups	9	47.591	0.005	0.14%	0.001(*F*_GT_)	0.012
	among individuals within group	592	2386.812	0.612	17.78%	0.178(*F*_IG_)	0.011
	within individuals	602	1703.219	2.829	82.08%	0.179(*F*_IT_)	0.016

A, B and C are three different grouping methods used in AMOVA; Method A treated samples from one site as a group, Method B divided all samples into urban and rural groups and Method C treated samples on the same side of a highway as a group. The subscripts of F refer to the hierarchical levels being compared; GT, groups to total population; IG, individual to group; individual to total population.

### Mantel test

Mantel tests on all samples, and on urban samples only, revealed no significant partial regressions between pairwise *F*_ST_ values and geographical distance, highway age, number of highways or the total number of roads between pairs of sample sites (Table 5). On the other hand, all partial regression *P* values for urban samples were lower than those for all samples,and all determination values of urban samples were higher than those for all samples (Table 5).

**Table 5:** Partial regression coefficients (b_Yj_) between *F*_ST_ values and geographical distance, age of highways, number of roads and number of highways respectively, *P* values for b_Yj_, and determination coefficients.

	samples	b_Yj_	*P*	Determination (%)
geographical distance	all samples	8×10^−6^	0.458	0.000
	urban samples	8×10^−5^	0.352	0.35
age of highways	all samples	3.5×10^−5^	0.601	0.001
	urban samples	5×10^−6^	0.492	0.002
number of roads	all samples	1.8×10^−5^	0.551	0.000
	urban samples	1×10^−6^	0.491	0.000
number of highways	all samples	1.8×10^−4^	0.302	0.007
	urban samples	8.7×10^−4^	0.097	4.98
% of variance explained by the model	all samples			0.008
	urban samples			5.132

## Discussion

As expected, the distance between sampling sites was too small for significant genetic differentiation among tree sparrows; the results of the Mantel test provide good support for the fact that there was no correlation between genetic differentiation and geographical distance. The Mantel test also indicated that there was no correlation between genetic variation and the number of highways, common roads and highway age. In addition, the AMOVA results also indicated very weak genetic variation between sites, between urban and rural populations, or between opposite sides of the same highway. Indeed, the data show that the genetic diversity of urban tree sparrows was not significantly different from those found at rural sites. These results do not, therefore, support the hypothesis that highways, or ordinary roads, significantly limit the dispersal of tree sparrows.

There was, however, some evidence to the contrary. We did find significant genetic differences between birds from 20% of sites, all of which were on opposite sides of the same highways, while no significant differences were found when sites were from the same side of the highway. This suggests that highways may be responsible for a weak genetic structure in urban tree sparrow populations. Furthermore, although we did not find any significant difference in the genetic diversity of urban and rural tree sparrow populations, we did find significant deviation from the Hardy–Weinberg equilibrium in some urban tree sparrow loci but no such deviation in birds from rural sites. This suggests that urban tree sparrows may be more inbred than those at rural sites. Meanwhile, the Mantel test returned lower *P* values and higher determination values when applied to urban samples than when applied to all samples, which suggests that highways have a greater effect on gene flow between urban than rural sites. These results suggest that highways, and especially urban highways, have a weak, but detectable, effect on gene flow in tree sparrow populations in Beijing.

There are several possible explanations for these results. The first is that tree sparrows can fly over roads, including highways. The second is population density. Gauffre et al. [37] demonstrated that genetic barrier effects are difficult to detect in species with large effective population sizes. The tree sparrow's high population density in parks, university campuses and suburban areas of Beijing [38] could, therefore, have reduced the likelihood of detecting genetic differences between sample sites. The third is temporal scale. The effects of genetic isolation typically develop over long periods of time [39], [40], but the oldest highway in this study was constructed in 1992 and had been in use for just 20 years. Because the tree sparrow is a relatively long-lived species [41], relatively few generations would have passed since these roads were built. We think it likely that Beijing's highways are too recent to have produced marked genetic differentiation in resident bird populations. In addition, highways with heavy traffic might have a bigger influence on the dispersal of birds than those with light traffic. High traffic volume is a relatively recent phenomenon in China where only 30 years ago private vehicles were rare compared to European or American cities of similar size. Therefore, Beijing's road network would be expected to have had less impact on gene flow in urban bird populations than the much older road systems in North America and Europe. All the above reasons could explain why we only detected relatively weak genetic effects.

Therefore, we cannot conclusively reject the hypothesis that highways do not restrict gene flow in urban tree sparrow populations. A time series analysis should be done to test whether the effects of highways on the genetic structure of Beijing's tree sparrow population will increase with time. Future research on the influence of highways on gene flow in bird populations will need to be conducted over several decades to obtain more conclusive results.
